# Diaphragmatic Pacing as an Initial Presentation of Delayed Ventricular Lead Perforation

**DOI:** 10.19102/icrm.2022.130504

**Published:** 2022-05-15

**Authors:** Luai Madanat, Kuldeep Shah, Richard Bloomingdale, Brian D. Williamson

**Affiliations:** ^1^Department of Internal Medicine, William Beaumont Hospital—Royal Oak, Royal Oak, MI, USA; ^2^Department of Cardiovascular Medicine, William Beaumont Hospital—Royal Oak, Royal Oak, MI, USA

**Keywords:** Cardiac implantable electronic device, coronary computed tomography angiography, diaphragmatic pacing, right ventricle perforation, transesophageal echocardiogram

## Abstract

Ventricular lead perforation is an infrequent and potentially fatal complication of pacemakers and implantable cardioverter-defibrillators that typically presents shortly following device implantation. Delayed lead perforations occurring 1 month after implantation are not widely reported and can have a wide range of presentations ranging from asymptomatic to potentially fatal cardiac tamponade. We describe a case of successful percutaneous lead extraction and revision in a patient who presented 9 months following implantation with an active fixation right ventricular pacing lead with apical perforation. Perforation was suspected when device interrogation showed ventricular sensing without ventricular capture, but with diaphragm stimulation. After an initial X-ray and transthoracic echocardiogram failed to detect it, computed tomography angiography confirmed the myocardial perforation. This case demonstrates the importance of recognizing such a complication following cardiac implantable electronic device implantation regardless of the timeline of presentation. It also serves to highlight the importance of clinical suspicion and awareness of the limitations of imaging for perforation. Transvenous percutaneous lead extraction and revision remains a favored approach due to reduced patient trauma when compared to the open surgical approach.

## Introduction

Myocardial lead perforation following cardiac implantable electronic device (CIED) implantation is an uncommon phenomenon and is reported to occur in 0.5%–2% of patients.^1^ Lead perforations typically occur within 24 hours of device implantations, and atrial leads are usually more involved.^2^ Delayed perforations, defined as those occurring >1 month later, remain rare; nevertheless, they are still reported in the literature.^2^ Patients with myocardial perforation can present with symptoms including chest pain, shortness of breath, palpitations, and pacemaker malfunction, or they can be asymptomatic with no obvious sign indicating disruption of myocardial integrity.^3^ Although uncommon, isolated diaphragmatic stimulation resulting from myocardial perforation has been reported in the literature.^4,5^ Several technical factors have been associated with an increased risk of lead perforation including active fixation of leads and the previous use of temporary wire pacing,^1^ which might explain why incidence is declining with the use of thinner, more flexible leads.^6^

## Case presentation

**Figure 1:**
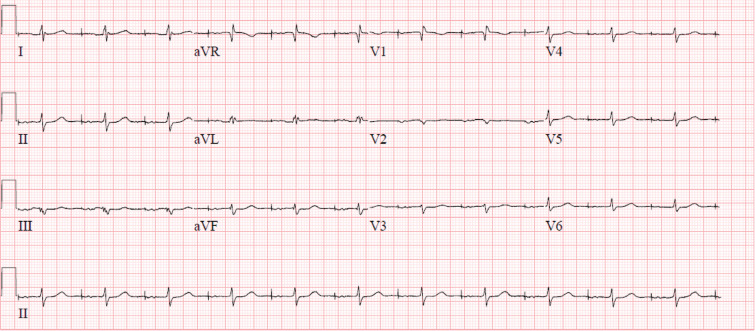
Electrocardiogram demonstrating an atrial paced rhythm with incomplete right bundle branch block.

**Figure 2:**
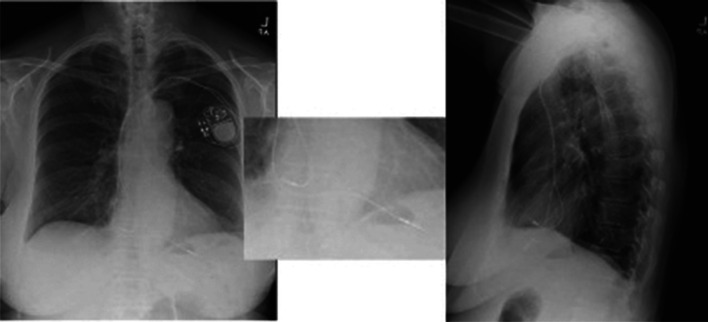
Chest X-ray at initial presentation. Anteroposterior and lateral view demonstrating evidence of pacemaker leads in the right atrium and right ventricle. A close-up of the pacemaker leads is shown in the center image.

**Figure 3:**
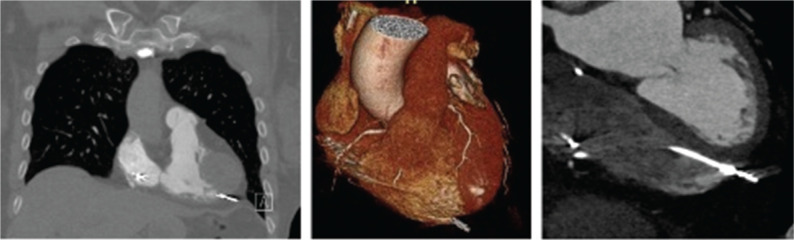
Computed tomography angiography demonstrating right ventricular lead perforation. A 3-dimensional reconstruction of the heart is shown in the center image. The tip of the lead is piercing the myocardium and extending outside the heart border.

**Figure 4:**
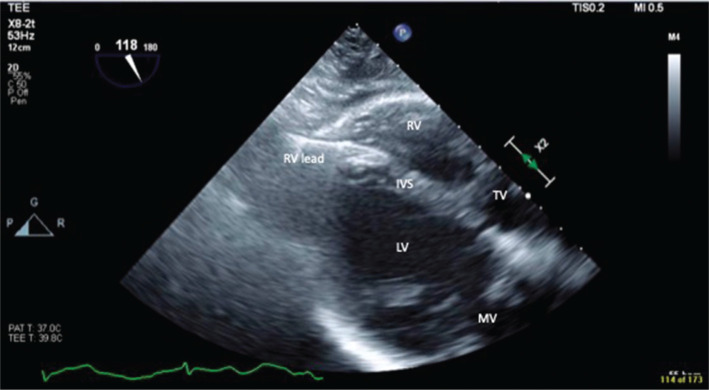
Intraoperative transesophageal echocardiography showing the right ventricular lead perforating through the apex. *Abbreviations:* IVS, interventricular septum; LV, left ventricle; MV, mitral valve; RV, right ventricle; TV, tricuspid valve.

We present the case report of a 77-year-old woman with a medical history of coronary artery disease, paroxysmal atrial fibrillation on anticoagulation, and sick sinus syndrome with a dual-chamber pacemaker (Medtronic, Minneapolis, MN, USA) implanted 9 months prior. She presented for a second opinion for the evaluation of intermittent palpitations and abdominal thumping sensation with visible abdominal wall contractions of 1 month’s duration. An electrocardiogram (ECG) demonstrated incomplete right bundle branch block with an atrial paced rhythm **(Figure 1)**. Device interrogation revealed stable right ventricular (RV) lead impedance and sensing but an inability to capture at 8 V at 1 ms with visible evidence of diaphragmatic stimulation. Previous cardiac device interrogation shortly following implantation showed a capture threshold of 2 V at 0.40 ms. She was subsequently sent for further workup based on altered device interrogation parameters. Chest X-ray (CXR) imaging was performed, which showed an apical RV lead, but within the cardiac silhouette, which was not diagnostic for a potential perforation through the apex **(Figure 2)**. Two-dimensional transthoracic echocardiography was performed, which demonstrated a left ventricular ejection fraction of 55% and a visible RV and right atrial lead with normal RV function. There was no evidence of pericardial effusion. Cardiac computed tomography (CT) angiography was performed. Despite the expected artifact from the pacing electrodes, which is always seen on CT imaging (“starburst”),^7^ it was possible to see the RV lead perforating through the myocardium **(Figure 3)**. The patient was scheduled for lead revision in a hybrid laboratory with cardiothoracic surgery backup. After opening the pocket, vascular access was first obtained and a new RV pacemaker lead was actively fixated to the apical interventricular septum. The old RV lead fixation screw was retracted, and the lead was removed without any resistance. There was no pericardial effusion present before, during, or after the procedure. Intraoperative transesophageal echocardiography (TEE) findings are demonstrated in **Figure 4**. It was only possible to see the perforated RV lead on TEE with non-standard views.

The patient was later transferred to the cardiac intensive care unit for postoperative monitoring. Post-procedure CXR did not reveal pleural effusion, and follow-up echo demonstrated minimal pericardial effusion with no evidence of tamponade. An ECG demonstrated adequately paced atrial rhythm with no new conduction abnormalities. Device interrogation was performed to ensure adequate parameters. The patient was subsequently discharged 72 hours after the procedure with appropriate follow-up scheduled.

## Discussion

Ventricular lead perforations following lead implantations in CIEDs are divided into acute (≤24 hours), subacute (5–29 days), and delayed (>30 days) cases according to their occurrence following implantation.^8^ Acute RV perforation may present with stabbing chest pain and shortness of breath and can often be associated with the development of pericardial effusion and cardiac tamponade.^9^ Delayed lead perforations, on the other hand, tend to present with lower rates of cardiac tamponade observed, probably due to the self-sealing properties of the ventricular wall by contraction, hemostasis, and subsequent fibrosis.^3^ Thus, late perforation could solely raise suspicion when pacing abnormalities ensue.^3^ This may in part explain the discrepancy observed between the incidence of RV perforation reported by studies. The Optim Lead Insulation Material (OPTIMUM) registry, aimed at monitoring the long-term outcomes of implantable cardioverter-defibrillator (ICD) and permanent pacemaker (PPM) leads, demonstrated the occurrence of RV perforation in 0.33% of ICDs versus 0.5% with PPM leads,^10^ while other reports revealed a prevalence of 6% upon CT evaluation of the patient.^7^

Several risk factors have been proposed to increase the risk of myocardial perforation, including recent steroid use, increasing age, female sex, and low body mass index.^11,12^ However, this may not preclude patients who do not fit the stereotype to have such complications.^13^ As previously stated, active fixation of screws is associated with higher rates of myocardial perforation.^6,7,14^ RV septal lead placement is presumed to entirely avoid perforations due to thick ventricular septal tissue. In a study of 2,247 lead implants, none of the cases of lead perforation involved septal leads.^15^ Conduction system pacing involving left bundle branch pacing and His-bundle pacing is gaining widespread interest in recent years due to the lower rates of pacing-induced cardiomyopathy and the ability to resynchronize left bundle branch block and presumably would be associated with fewer perforations.^16^

Although suspicion for lead perforations can arise based on clinical grounds and device interrogations as demonstrated in our case, confirmation of myocardial perforation is established by imaging demonstrating the lead extending beyond the contour of the heart. CXR images are often initially obtained and can grossly demonstrate the position of the lead; however, cases of subtle perforations are often missed due to the inability of plain radiography to differentiate between the vascular cavity, myocardium, and pericardium.^3,17^ Transthoracic echocardiography is often crucial to evaluate for the presence of pericardial effusion and can help visualize the position of the lead in cases of myocardial perforations.^18^ However, depending on the spatial orientation of the echocardiography beam, it may not reliably establish the lead position and can prove to be non-diagnostic in some cases.^5,19^ Three-dimensional echocardiography has been proposed to be superior in lead visualization as it provides a more comprehensive view of the intracardiac structure.^19^ Cardiac CT has been considered the gold standard in the diagnosis of myocardial lead perforation.^20^ In a retrospective study involving 426 participants, cardiac CT was shown to be the method of choice in diagnosing myocardial perforations and demonstrated higher sensitivity compared to echocardiography (100% vs. 41.2%, respectively).^21^ Magnetic resonance imaging (MRI) has not been traditionally used due to potential catastrophic effects in interactions with intracardiac devices.^22^ With the advent of new MRI-compatible devices, however, the utilization of MRI could be of value considering its fewer artifacts compared to CT.^3^

Proposed management of late lead perforations has been unresolved in the literature. Lead extraction in asymptomatic patients has been suggested to be unnecessary with additional lead implantation sufficient to solve the problem. However, this entails an avoidable risk of lead migration and the potential for further damage.^23^ In cases where tamponade has developed or the patient is symptomatic, treatment is warranted, with previous literature recommending and demonstrating favorable outcomes with transvenous percutaneous extraction with the utilization of TEE guidance as an additional safety precaution.^13,23^

In the present case, cardiac device interrogation shortly following implantation was unremarkable with no evidence of lead malfunction. She started experiencing symptoms approximately 1 month prior to presentation, which could indicate the onset of myocardial perforation. Similar to previous reports,^13^ initial routine imaging including chest radiographs and transthoracic echocardiography failed to demonstrate the defect. PPM interrogation demonstrated device malfunction, which subsequently raised the suspicion for possible myocardial perforation. However, normal pacing and sensing parameters should not exclude the diagnosis.^7^ The diagnosis was later confirmed with CT angiography.

## Conclusion

Late RV perforation remains an uncommon complication of CIED implantation. Diagnosis can often be suspected based on clinical presentation and device parameters and should be confirmed with imaging. Cardiac CT has been shown to be an excellent screening tool and confirmatory test to establish diagnosis. Transvenous percutaneous lead extraction and revision remain a favored approach due to reduced patient trauma compared to the open surgical approach. However, it is prudent to have surgical backup in case unforeseen complications ensue.^24^
